# Preventive Strategies for Upper Extremity Deep Venous Thrombosis Following Elective Upper Limb Surgery: A Systematic Review

**DOI:** 10.3390/clinpract15120221

**Published:** 2025-11-26

**Authors:** Aeshah Salem Alsharidah, Alya Ali Aljubran, Maha Alkharisi, Taif Alnafie, Dhai Almuteri, Zahra Almarhabi, Noor Alawami, Shaykhah Alkulaib, Hashmiah Aljarash, Zain Abdullah, Abdullah Almaqhawi

**Affiliations:** 1 King Abdulaziz Hospital, Ministry of National Guard Health Affairs, Al Ahsa 36428, Saudi Arabia; dr.alsharidah1@gmail.com (A.S.A.); shikahsultan2@gmail.com (S.A.); 2 College of Medicine, King Faisal University, Al Ahsa 31982, Saudi Arabia; alya.aljubran@outlook.sa; 3 College of Medicine, Imam Mohammad Ibn Saud Islamic University, Riyadh 13317, Saudi Arabia; alkhrisimaha@gmail.com; 4 College of Medicine and Surgery, King Abdulaziz University, Jeddah 21589, Saudi Arabia; tafealnafei@gmail.com; 5 King Fahad Specialist Hospital, Qassim Health Cluster, Al-Qassim, Buraydah 52366, Saudi Arabia; dhayzabar1@gmail.com; 6 College of Medicine and Surgery, Umm Al-Qura University, Alqunfudah 21955, Saudi Arabia; zahra.abdu30i@gmail.com; 7 Family Medicine Academy, Eastern Health Cluster, Dammam 32253, Saudi Arabia; nooralawami9@gmail.com; 8 College of Medicine and Surgery, Dar Aluloom University, Riyadh 13314, Saudi Arabia; 9 Fakeeh College for Medical Sciences, Jeddah 21461, Saudi Arabia; zain.jaabdullah@hotmail.com; 10 Department of Family and Community Medicine, College of Medicine, King Faisal University, Al-Ahsa 31982, Saudi Arabia

**Keywords:** upper extremity deep vein thrombosis (UEDVT), thromboprophylaxis, deep vein thrombosis, elective upper limb surgery

## Abstract

**Background/Objectives:** Upper extremity deep vein thrombosis (UEDVT) is a harmful complication of elective upper limb surgeries. Different strategies are employed to prevent this condition. The aim of the review is to quantify the effectiveness of various preventive interventions and investigate correlated factors that affect the incidence of UEDVT (upper extremity deep vein thrombosis). **Methods**: We performed a systematic search using the PubMed, EBSCO, Ovid, EMBASE, Cochrane, and Google Scholar databases. Randomized controlled trials (RCTs), prospective or retrospective cohort studies, or case–control studies were examined. We included adult patients over 18 years old undergoing elective upper limb surgery and receiving Prophylactic measures for Upper Extremity Deep Venous Thrombosis. **Results**: After a literature search and quality assessment, 6 studies were included. All the studies were of good quality but significantly heterogeneous in terms of sample size, population size, treatment modalities, and baseline characteristics. In these studies, the reported incidence of symptomatic venous thromboembolism (VTE) varied widely, ranging from 0.41% to 13%. However, thromboprophylaxis did not have a significant impact on the rates of deep vein thrombosis (DVT). Certain factors such as older age and trauma as the cause of surgery were identified as notable risk factors for symptomatic VTE. **Conclusions**: This systematic review highlights the complexity of preventing upper extremity deep vein thrombosis (UEDVT) following elective upper limb surgeries. The reported incidence of symptomatic VTE varies considerably across studies, and thromboprophylaxis was not associated with a significant reduction in its rates. The evidence is characterized by substantial heterogeneity in patient populations and surgical contexts. More research is needed to better understand the role of thromboprophylaxis in preventing DVT.

## 1. Introduction

Upper extremity deep vein thrombosis (UEDVT) is a condition characterized by the formation of a blood clot (thrombus) within the deep veins of the upper limb. ULDVT is classified as primary or secondary. Primary ULDVT, which includes conditions like Paget-Schroetter syndrome (effort thrombosis), is rare and often related to anatomical variations or repetitive venous trauma. The reported incidence of symptomatic deep vein thrombosis and pulmonary embolism following elective upper limb surgery is less than 0.20%, which is low compared to the average of 0.66% for lower limb alternatives [1,2]. It can lead to the development of pulmonary thrombosis and other severe outcomes, making prevention methods important [3].

Surgical operations on the upper extremities are connected with an increased risk of DVT. In the postoperative period, 5.7% of patients experience the asymptomatic form, 0.20%—pulmonary embolism, and 0.22% of the cases may result in death [2,3]. At the same time, academic literature does not provide specific prevention techniques, with most articles focusing on the lower extremities.

Given the variation in practice patterns and the lack of definitive guidelines regarding thromboprophylaxis in elective upper limb surgery, a systematic review of the available literature is necessary. Understanding which patients are at the highest risk of UEDVT and identifying the most effective preventive strategies can help optimize perioperative management and reduce complications. Existing research has explored various thromboprophylaxis measures, including mechanical prophylaxis (e.g., early mobilization, compression devices) and pharmacological interventions (e.g., low-molecular-weight heparin, direct oral anticoagulants), yet there is no consensus on the most effective approach. Furthermore, risk factors such as older age, history of thrombosis, prolonged surgical duration, and trauma-related surgeries have been inconsistently reported across studies.

Several key features of the articles on the topic support the rationale behind the research. While the prevalence of upper limb DVT is relatively lower, the awareness of the complication is inadequate, and preventive measures are non-specific [4]. The existing works mention that the current strategy aims to decrease the risk of all types of thromboembolic conditions [5]. This gap in practice leads to worse diagnosis rates and an increased percentage of asymptomatic yet impactful cases. Although the mortality risk of upper extremity thrombosis is lower than that of thrombosis in other locations, it may lead to long-term adverse effects. Moreover, the last significant changes of the 2007 National Institute for Health and Clinical Excellence guidelines did not result in noticeable improvements in preventive measures [2]. Therefore, closing this gap will increase the effectiveness of surgery and postoperative units by improving upper limb venous thrombosis strategies.

This systematic review aims to systematically identify, evaluate, and synthesize the current evidence regarding DVT prevention strategies after elective upper limb surgery. The expected outcome is a comprehensive summary of the available evidence on the efficacy and safety of these measures, which will serve as a foundation for clinical decision-making and guideline development.

## 2. Materials and Methods

This systematic review was prospectively registered in PROSPERO (CRD42023455186) and conducted following the Preferred Reporting Items for Systematic Reviews and Meta-Analysis (PRISMA) guidelines [6].

### 2.1. Participants

This review encompassed studies involving adults aged 18 and older who underwent elective upper extremity surgery and received DVT prophylaxis. Eligible studies were required to report the incidence of UEDVT and associated complications or the preventive strategies employed. Acceptable study designs included randomized controlled trials (RCTs), prospective or retrospective cohort studies, or case–control studies. Furthermore, studies needed to be published in English.

Exclusion criteria comprised abstracts, editorials, letters, case reports, and reviews. Studies with a high risk of bias or those not investigating UEDVT, reporting its incidence or complications, were also excluded.

### 2.2. Literature Search

We systematically searched PubMed, EBSCO, Ovid, EMBASE, Cochrane, and Google Scholar using predefined search criteria and terms. The search strategy, devised following the traditional PICO method, incorporated Medical Subject Headings (MeSH) to develop a comprehensive search strategy. Keywords such as “preventive strategies”, “upper extremity”, “deep vein thrombosis”, and “post-exposure prophylaxis” were included. The aim was to identify allrelevant studies focusing on prophylaxis for upper extremity deep venous thrombosis following elective upper limb surgery. The full research strategy is available in the supplemental information). To ensure comprehensiveness, a supplementary manual search was conducted by hand-searching reference lists of included articles and key journals in the medical field. The same predefined criteria used for electronic database searches were applied during the manual search. The initial search results were imported into Mendeley for deduplication. The deduplicated results were subsequently imported into Rayyan, and screened by four authors (T.A., A.A., Z.A., Z.A.) based on titles and abstracts. For final inclusion or exclusion, the full texts from both electronic and manual searches underwent independent screening by four authors (T.A, D.A., N.A., Z.A.). Any discrepancies during the screening process were resolved through discussion and consensus among all authors.

### 2.3. Data Extraction and Quality Assessment

Data extracted from the retained studies included title, first author’s name, journal name, year of publication, country of origin, study design, sample size, demographic details (age, gender), procedural details (laterality, DVT risk factors, operative procedure), preventive strategies (intervention, pharmacological, mechanical, others), duration of follow-up, UEDVT incidence, complications related to preventive strategies or UEDVT, main findings, and limitations.

## 3. Results

### 3.1. Study Selection

**Figure 1:**
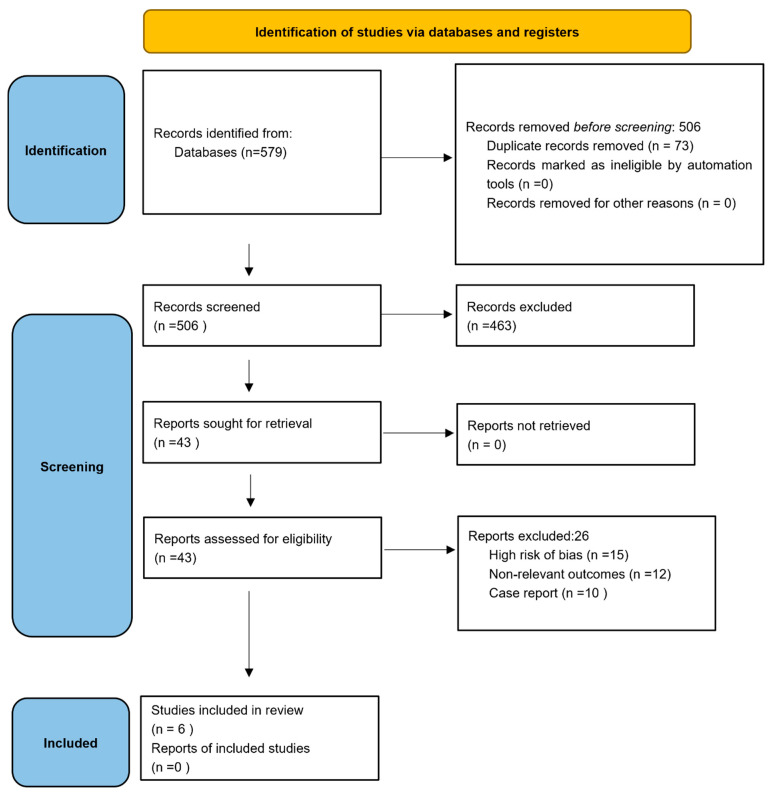
A PRISMA flow diagram.

The initial systematic search across PubMed, EBSCO, Ovid, EMBASE, Cochrane, and Google Scholar yielded 579 articles. After removing 73 duplicates, 506 records underwent title and abstract screening. Of these, 463 were excluded for not meeting inclusion criteria. The remaining 43 full-text articles were assessed for eligibility. Finally, six studies met the inclusion criteria, comprising two retrospective cohort studies, two prospective cohort studies, and two non-randomized comparative studies [1,2,3,4,7,8]. No randomized controlled trials (RCTs) were identified. A PRISMA flow diagram is provided in Figure 1, and the PRISMA checklist is available in the Supplementary Material.

### 3.2. Study Characteristics

The six included studies, published between 2009 and 2022, involved 8516 patients undergoing elective upper limb surgeries, including shoulder arthroplasty, arthroscopic rotator cuff repair, and proximal humeral fracture surgery. The smallest sample size was of 57, while one study was a large database study. Key risk factors included advanced age (>60 years, reported in 4/6 studies), obesity (BMI > 30 kg/m^2^, 3/6 studies), smoking (prevalence: 45–70%, 2/6 studies), and comorbidities such as hypertension (63–80%, 2/6 studies) and diabetes (20–30%, 2/6 studies).

### 3.3. Preventive Strategies

Prophylactic measures were reported with limited consistency. Three studies utilized low-molecular-weight heparin (LMWH) postoperatively, though dosing regimens were unspecified. One study administered 81 mg/day aspirin for four weeks [1,7,8]. Two studies did not report pharmacological prophylaxis [3,4]. Intraoperative pneumatic compression devices were used in one study [3], while postoperative elastic stockings and early mobilization were employed in another. Two studies combined mechanical and pharmacological methods. Three studies included cohorts without prophylaxis, though selection criteria for these groups were not standardized.

### 3.4. DVT and Complications

The incidence of UEDVT varies significantly, reported between 0.51% and 13%, largely attributable to variations in study design and case definitions. Navarro et al. [4] reported a 0.51% incidence of symptomatic upper extremity deep vein thrombosis (7/1574) in patients with implantable cardioverter-defibrillators, with diagnosis determined by clinical presentation. Willis et al. [7] reported a significantly higher incidence of 13% (9/70) in patients with peripherally inserted central catheters. This rate was determined through systematic, protocol-driven Doppler ultrasound screening, capable of identifying both symptomatic and asymptomatic cases. This demonstrates that screening-based studies identify a wider, frequently subclinical, disease burden in contrast to studies that depend on symptomatic presentation. Takahashi et al. [3] identified a 5.7% rate (10/175) via ultrasound, predominantly in the axillary and brachial veins. Incidences ranged from 0.54% (Navarro et al.) to 6% (Willis et al.), highlighting the systemic nature of thromboembolic risk (Takahashi H et al., Navarro RA et al., Willis AA et al.) [3,4,7]. (Table 1 and Table 2).

Pulmonary embolism (PE) occurred in 0.2–1.4% of cases, with one fatal PE reported. Complications from prophylaxis included bleeding events (*n* = 3, Alyea et al.) and gastrointestinal irritation (*n* = 2, Alyea et al.) [1] (Table 3).

Advanced age (>70 years) and traumatic surgical indications were consistently associated with higher UEDVT risk. Extreme obesity (BMI > 50 kg/m^2^, corresponding to Class IV obesity) tripled VTE risk in one study (Koch et al.) [8], while smoking and cancer history showed non-significant trends (Table 4).

### 3.5. Study Heterogeneities

Substantial clinical and methodological heterogeneity precluded meta-analysis. Therefore, a narrative synthesis was performed to present the findings. Variability arose from differences in operative duration and positioning. Similarly, only 65% of patients in the two studies underwent postoperative ultrasound; others relied on symptomatic reporting. The follow-up duration ranged from 12 weeks to 5 years, with longer follow-up correlating with higher DVT detection. The follow-up duration ranged from 12 weeks to 5 years, with longer follow-up correlating with higher DVT detection.

Study limitations included small sample sizes (n < 100 in 3/6 studies), single-center designs (4/6 studies), and retrospective data collection (2/6 studies). Only one study reported using validated tools (Caprini score) for risk stratification [9].

The quality of the included studies was assessed independently using the MINORS tool by three authors for retrospective and prospective cohort studies (Table 5) [10].

Despite diligent efforts, conducting a basic descriptive statistical analysis posed challenges due to significant heterogeneity among the included articles and data in an unsuitable format for meta-analysis (Table 6). Consequently, a meta-analysis was not performed due to significant clinical and methodological heterogeneity, evaluated both conceptually and statistically. The conceptual assessment took into account differences in patient groups, treatments (like the length of the surgery and the way the patient was positioned), and outcome measures (like confirming DVT with an ultrasound versus reporting symptoms and having follow-ups that lasted anywhere from 12 weeks to 5 years). The expected statistical heterogeneity (I^2^ statistic), had a meta-analysis been conducted, was assessed to be substantial due to these clinical and methodological disparities.

## 4. Discussion

The reported incidence of symptomatic VTE across the included studies varied widely, from 0.41% to 13%. Furthermore, the analysis indicated that thrombo-prophylaxis did not have a significant effect on the rate of DVT. Age and surgeries pertaining to trauma represent major risk factors. The current research about UEDVT prevention has demonstrated the necessity for ongoing studies to improve prophylactic methods. Our research simplifies existing evidence through a review of preventive methods along with patient-based risk factors which impact thrombosis.

Delluc et al. documented UEDVT has an estimated incidence of 0.4–1 case per 10,000 persons, which indicates thromboprophylaxis can be selectively applied to surgical interventions on upper limbs because of the very low risk of venous thromboembolism [9]. Alizadehasl et al. presented through their review study that thromboprophylaxis delivers beneficial outcomes when provided to high-risk patients. They found that pharmacologic interventions using low-molecular-weight heparin (LMWH) demonstrated a substantial decrease in thrombotic occurrences when used for patients who possess risk variables such as malignancy or previous thrombotic events [11]. The conflicting results between studies could result from various selection methods used for patients, prophylaxis plans as well as surgical approaches.

The findings of Smith et al. support the notion that trauma functions as a risk factor for symptomatic VTE and matches the results of this study. Through their study, they found that injuries to the endothelial cells following trauma significantly increased the chance of postoperative clot formation [12]. The analysis conducted by Sheth et al. showed that patients’ post-operative immobility stood as the main risk factor instead of traumatic injuries [13]. These findings show that trauma likely starts the thrombotic cascade but immobility after surgery becomes a greater factor in clot progression [14,15]. Thus, personalized risk evaluations together with specific preventive measures need to be implemented during surgical procedures that involve trauma patients.

This study’s findings about the minimal effectiveness of thromboprophylaxis techniques match the results described by Badr et al. who showed that DVT prevention medications failed to lower venous clot rates. Their analysis showed that UEDVT’s risk was too minimal for medication-based prevention to generate noticeable impact on every patient [16]. In contrast, the application of pharmacological thromboprophylaxis delivered major VTE protection for orthopedic patients during joint replacement surgery as well as extensive tissue surgical procedures per Panamsky et al. [17,18]. The conflicting viewpoints about patient response rates according to surgical subtypes and individual characteristics underline the necessity of creating distinct preventive guidelines in anticoagulation.

Studies on VTE risk factors demonstrate that old age results in higher thromboembolic risks including DVT according to Cowan et al. as reported in surgical patients. The natural changes that occur in coagulation along with vascular integrity in elderly patients establish a higher risk of blood clots [19]. However, some studies have revealed that age alone does not influence VTE risk because cardiovascular diseases, diabetes and obesity prove to be greater factors behind VTE development according to Tsai et al. [20]. Thus, decision-making regarding thromboprophylaxis requires patient evaluation beyond age-specific factors because it supports risk assessment through multimodal tools.

Research by Urbankova et al. shows intermittent pneumatic compression (IPC) as one example of mechanical prophylaxis that proves ineffective for UEDVT. The authors pointed out that IPC devices work well for lower limb surgeries [21], yet the upper extremity benefits remain uncertain because of anatomical and blood flow differences. The combination of pharmacologic with mechanical methods such as IPC and graduated compression, proved exceptionally effective for preventing lower limb DVT according to the research by Langridge et al. [22]. These findings suggest that clot formation occurs differently between upper and lower extremities thus warranting more investigation to optimize mechanical preventive methods used in upper limb surgeries.

This review’s strengths include the comprehensive inclusion of observational and clinical trial data, providing a well-rounded perspective on the topic. Adherence to systematic review protocols enhances the reliability of our conclusions. The heterogeneity of included studies in our review is a limitation, as variations in sample size, methodology, and patient populations may affect the generalizability of our findings. Differences in prophylactic protocols used in included studies contribute to inconsistencies in reported outcomes.

More research should be conducted to develop extensive multicenter clinical trials for testing thromboprophylaxis treatments specifically among different patient categories. The development of new preventive methods should be pursued which would involve creating individualized prophylaxis plans based on each patient’s risk factors. Future research on UEDVT prevention in elective upper limb surgery needs to establish definitive protocols for best practices of preventive measures. Studies investigating both new pharmacologic medications with better safety characteristics and genetic risk factors for thrombosis should aim to establish personalized approaches for thrombo-prophylaxis.

## 5. Conclusions

This systematic review highlights that the current evidence regarding upper extremity deep vein thrombosis (UEDVT) following elective upper limb surgery remains limited, heterogeneous, and of moderate methodological quality. Reported incidence rates vary markedly, largely due to inconsistencies in study design, screening protocols, and follow-up duration. Advanced age, obesity, and traumatic surgical indications appear to be consistent risk factors, whereas the roles of smoking and cancer history remain insufficiently defined. Preventive strategies—including pharmacological and mechanical approaches—were inconsistently applied, and standardized prophylactic regimens were rarely reported.

Taken together, these limitations require cautious interpretation of the available evidence. Clinicians should maintain vigilance in high-risk surgical populations and consider individualized prophylactic strategies where appropriate. The absence of consensus also underscores the urgent need for well-designed prospective studies. Future research should prioritize the development and validation of standardized risk-stratification tools, directly compare mechanical and pharmacological prophylaxis, and incorporate randomized controlled trials to establish evidence-based preventive protocols. Strengthening the quality of research in this domain is essential to guide clinical decision-making and enhance patient safety in elective upper limb surgery.

## Figures and Tables

**Table 1:** Statistical techniques, complications associated with DVT in the upper and lower extremities, and complications related to preventive measures.

Complications Related to Preventive Strategies				Complications Related to Upper Extremities DVT				Complications Related to Lower Extremities DVT				Statistical Methods Used	
Group 1	Total	Group 2	Total	Group 1	Total	Group 2	Total	Group 1	Total	Group 2	Total		Authors
NM	NM	NM	NM	14 (PE) no mention to the site of origin	NM	NM	NM	NM	NM	NM	NM	not specifically mentioned	Navarro [4]
NM	NM	NM	NM	1 (PE)	1	NM	NM	NM	NM	NM	NM	X2 analysis or the Fisher exact test	Takahashi [3]
not specifically mentioned	NM	NM	NM	not specifically mentioned	NM	NM	NM	not specifically mentioned	NM	NM	NM	not specifically mentioned	Jameson [2]
Bleeding events, peptic ulceration, and gastric irritation, dyspepsia, hematoma	NM	NM	NM	1 (PE)	NM	0 (PE)	NM	1 (PE)	NM	0 (PE)	NM	SAS 9.4 Platform, (IBM) Wilson methods, Newcombe method, Firth method	Alyea [1]
NM	NM	NM	NM	NM	NM	NM	NM	NM	NM	NM	NM	NM	Koch [8]
NM	NM)	NM	NM	1 (fatal PE), 2 (non-fatal PE)	3	NM	NM	NM	NM	NM	NM	SPSS (version 31) computer software	Willis [7]

Note: NM = Not Mentioned. References: Alyea et al. (2019) [1]; Jameson et al. (2011) [2]; Takahashi et al. (2014) [3]; Navarro et al. (2013) [4]; Willis et al. (2009) [7]; Koch et al. (2017) [8].

**Table 2:** Comparison of VTE Risk Factors Across Shoulder Surgery Studies.

Laterality		VTE Risk Factors		VTE Risk Factors		Operative Procedure		
Right	Left	Group 1	Total	Group 2	Total	Group 1	Group 2	Authors
1474	1100		NM	NM	NM	RSAs, TSAs, HAs	NM	Navarro [4]
NM	NM	(non-DVT group): mean age 60 ± 14, male: female ratio 2.4:1, mean BMI 24.3 ± 3.3, mean operative time 135 min, with smoking habit 45%, with DVT risk-related comorbidity 63%	165	(DVTgroup): mean age 66 ± 7, male: female ratio 4:1, mean BMI: 24.3 ± 3.8, mean operative time 139 min, with smoking habit 70%, with DVT risk-related comorbidity (HTN, DM, prostate cancer) 80%.	10	ARCR, A-patch, ABR, other	NM	Takahashi [3]
NM	NM	those aged over 60 years, obese, Surgical procedure (those undergoing operations where the total combined anesthetic and surgical time is greater than 90 min), Patient comorbidities	NM	NM	NM	Total Shoulder Replacement, Arthroscopy, Proximal Humeral Fracture Surgery	NM	Jameson [2]
564	350	old age, revision surgery, smoking and cancer history, history of PE and high cholesterol and blood pressure inherited and acquired hypercoagulable states.	NM	old age, revision surgery, smoking and cancer history, history of PE and high cholesterol and blood pressure inherited and acquired hypercoagulable states.	NM	PCR	PCR	Alyea [1]
NM	NM	the patients with a BMI of >50 kg/m^2^ have three times higher risk of VTE than the non-obese.	NM	NM	NM	compression and Doppler ultrasound	NM	Willis [7]
NM	NM	Age has been shown to be significant risk factor for VTE disease.	NM	NM	NM	standard deltopectoral approach	NM	Koch [8]

Note: NM = Not Mentioned. References: Alyea et al. (2019) [1]; Jameson et al. (2011) [2]; Takahashi et al. (2014) [3]; Navarro et al. (2013) [4]; Willis et al. (2009) [7]; Koch et al. (2017) [8].

**Table 3:** Summary of Mechanical and Pharmacological Preventive Strategies for VT In Surgical Patients.

Preventive Strategies (Pharmacological)			Preventive Strategies (Mechanical Prophylaxis)		
Dosage	Timing of Administration	Mode of Delivery	Dosage	Timing of Administration	
no preventive strategies were mentioned.	NM	NM	NM	NM	Navarro [4]
pneumatic compression devices	During surgery	NM	elastic stocking	During surgery	Takahashi [3]
chemical thromboprophyl axis (LMWH prophylaxis)	NM	NM	not specifically discussed	NM	Jaameson [2]
81 mg/d aspirin for 4 weeks	postoperatively	NM	compression boots and early mobilization	postoperatively	Alyea [1]
thromboprophyl axis (LMWH prophylaxis)	NM	NM	NM	NM	Koch [8]
Postoperative thromboprophyl axis with pharmacologic agents is widely advocated to improve survival rates and reduce health care costs	NM	NM	NM	NM	Willis [7]

Note: NM = Not Mentioned. References: Alyea et al. (2019) [1]; Jameson et al. (2011) [2]; Takahashi et al. (2014) [3]; Navarro et al. (2013) [4]; Willis et al. (2009) [7]; Koch et al. (2017) [8].

**Table 4:** Main Findings and Reported Confidence Intervals of Studies Reporting VT in shoulder surgery.

Confidence Intervals	The Study’s Main Findings	Authors
95%	No statistically significant associations between procedure type or surgery indication and odds of PE, DVT, VTE, or 90-day mortality were observed. incidence of symptomatic DVT 0.51% and 0.54% for symptomatic PE for all patients with primary shoulder arthroplasties.	Navarro [4]
95%	incidence of asymptomatic DVT after surgery: 5.7%. symptomatic PE: 0%. DVT occurred at 1 or 2 days post-op	Takahashi [3]
95%	Low incidence of symptomatic VTE (0.41%). Risk factors included age > 70 years, traumatic indication. VTE led to prolonged hospital stay and readmission	Jaameson [2]
95%	VTE) after shoulder surgery are relatively rare. The implementation of national thromboprophylaxis guidelines did not significantly impact the rates of VTE after shoulder surgery	Alyea [1]
95%	In a study involving 57 participants who underwent various types of shoulder replacements, the incidence of deep vein thrombosis (DVT) was found to be 12.3% (7 out of 57) with a confidence interval of 95% [5.1, 23.7]. Two DVTs were observed in the upper limb, while two others occurred in the lower leg. All DVTs were reported to be of acute onset, with two causing partial vein obstruction. The cardiology department managed the treatment, and symptomatic DVTs were observed in one patient with axillary vein DVT and one with brachial vein DVT. Four participants were already on thromboprophylaxis at the time of diagnosis. No fatalities were reported. There was no significant association between smoking and DVT risk. Females with reverse shoulder replacements had longer post-operative stays. The average Caprini score was 5.8, with higher scores in individuals with positive duplex Doppler, females with DVT, and males having lower scores. Among participants with shoulder replacements due to trauma, one had a DVT despite receiving thromboprophylaxis. The average BMI was 32.1, and the mean age was 62.6 years. The duration of surgery did not differ significantly between participants with and without DVT. One participant had a previous DVT history and developed a symptomatic axillary vein DVT post-surgery.	Koch [8]
95%	In this study on shoulder arthroplasty, the prevalence of deep vein thrombosis (DVT) was found to be 13%. Surveillance Doppler ultrasound conducted on the second postoperative day detected 10 DVTs (prevalence of 10%) in 9 patients, and an additional 3 new DVTs (incidence of 6%) were documented in 3 patients 12 weeks after surgery. DVTs were observed in both the upper and lower extremities during the acute and subacute postoperative periods. Upper extremity DVTs were exclusively found in the operative extremity, with involvement of the axillary and brachial veins. Lower extremity DVTs included ipsilateral and contralateral cases, involving the popliteal, posterior tibial, and peroneal veins. Three patients experienced pulmonary embolism, with 2 cases confirmed by spiral CT scan. One patient died due to cardiovascular collapse from a massive pulmonary embolism. The prevalence of DVT in this study was significantly higher than that in the general population and age-matched control subjects, but not significantly different from the prevalence after hip replacement surgery. However, the prevalence was significantly lower than that after knee replacement surgery.	Willis [7]

References: Alyea et al. (2019) [1]; Jameson et al. (2011) [2]; Takahashi et al. (2014) [3]; Navarro et al. (2013) [4]; Willis et al. (2009) [7]; Koch et al. (2017) [8].

**Table 5:** MINORS Tool for Selected Non-Randomized Studies.

Willis et al. (2009) [7]	Takahashi et al. (2014) [3]	Navarro et al. (2013) [4]	Koch et al. (2017) [8]	Jameson et al. (2011) [2]	Alyea et al. (2019) [1]	
2	2	2	2	2	2	A clearly stated aim
1	2	2	2	2	2	Inclusion of consecutive patients
0	2	2	2	2	1	Prospective collection of data
1	0	2	2	1	2	Endpoints appropriate to the aim of the study
0	0	0	0	0	0	Unbiased assessment of the study endpoint
1	2	1	0	1	2	Follow-up period appropriate to the aim of the study
0	0	2	1	0	0	Loss to follow-up less than 5%
0	2	2	2	2	2	Prospective calculation of the study size
Additional criteria in the case of comparative study:						
1	1	0	-	-	2	An adequate control group
0	2	0	-	-	2	Contemporary groups
1	2	2	-	-	2	Baseline equivalence of groups
2	2	2	-	-	2	Adequate statistical analyses
9/24	17/24	17/24	11/16	10/16	19/24	Total (out of 16 or 24)

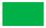
High quality, 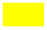
Moderate quality, 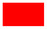
poor quality.

**Table 6:** Recommendations for Future Research and Study limitations.

Limitations	Recommendations for Future Research	Authors
Not all patients underwent screening (with ultrasound, venography, or CT) either before or after the surgery. describing only the symptomatic cases of VTE. unable to evaluate the associations of many risk factors and incidence of VTE. the incidence of DVT is probably higher than reported. it still may be underpowered to detect the 0.9% observed difference in VTE and mortality rates between elective and traumatic arthroplasties.	The data in this study may be considered hypothesis-generating and useful for sample-size calculations in future prospective studies regarding routine VTE prophylaxis in the shoulder arthroplasty population.	Navarro [4]
lateral decubitus position was not included, DVT prophylaxis was not controlled, US was performed in only 85 patients at 3 weeks to 3 months after surgery. ultrasound examination for patients who had total knee arthroplasty was performed at 3 days after surgery or later. ex post analysis of the statistical power for DVT were lower than 0.4.	prospective studies including greater numbers of patients are needed to determine the risk factors	Takahashi [3]
not all patients underwent screening (with ultrasound, venography, or CT) either before or after the surgery. describing only the symptomatic cases of VTE. unable to evaluate the associations of many risk factors and incidence of VTE. the incidence of DVT is probably higher than we reported. it still may be underpowered to detect the 0.9% observed difference in VTE and mortality rates between elective and traumatic arthroplasties.	the data in this study may be considered hypothesis-generating and useful for sample-size calculations in future prospective studies regarding routine VTE prophylaxis in the shoulder arthroplasty population.	Jaameson [2]
selection bias and lack of randomization. Small sample size: The study included 914 patients, with 430 in the aspirin group and 484 in the control group. Low event rate: The overall incidence of VTE events was very low, with only 7 events (6 DVTs and 1 PE) occurring during the study period. This low event rate makes it difficult to draw definitive conclusions about the effectiveness of aspirin in preventing VTE. Lack of standardization: The study did not report on the specific criteria or protocols used for VTE screening and diagnosis. This could have led to variability in the detection and reporting of VTE events. Limited generalizability: The study was conducted at a single institution, which may limit the generalizability of the findings to other populations or settings. Outdated data: The study included patients treated between 2010 and 2015, which may not reflect the current standard of care or the use of newer VTE prophylaxis strategies.	A larger sample size would have increased the statistical power to detect any meaningful differences between the groups. Conduct a prospective, randomized controlled trial would provide more robust evidence on the effectiveness of aspirin for VTE prevention in patients undergoing arthroscopic rotator cuff repair. Standardize VTE screening and diagnosis: Establish clear, standardized protocols for the assessment and diagnosis of VTE events. Assess long-term outcomes	Alyea [1]
Small sample size (57 participants), Conducted at a single institution, Potential selection bias in participant inclusion, Lack of a control group for comparison, Insufficient information on statistical methods used, Follow-up duration not specified.	Conduct studies with larger sample sizes to enhance statistical power and generalizability. Include multiple healthcare institutions to capture diverse patient populations and account for variations in surgical techniques and postoperative care. Design randomized controlled trials with control groups to compare DVT incidence against baseline rates. Provide detailed reporting of statistical methods used in the analysis. Conduct studies with long-term follow-up to assess the incidence of DVT and potential complications over time. Investigate risk factors associated with DVT after shoulder replacement surgery to identify high-risk subgroups. Evaluate the effectiveness of thromboprophylaxis measures in reducing DVT incidence.	Koch [8]
The study presented several limitations that should be considered when interpreting the findings. Firstly, the sample size was small, which may limit the statistical power and generalizability of the results. Secondly, the study was conducted at a single center, further reducing the generalizability to other settings. Thirdly, the study employed an observational design, which prevents the establishment of causal relationships between variables. Fourthly, certain patient groups were excluded from the study, introducing potential selection bias and limiting the applicability of the findings to a broader population. Finally, the relatively short follow-up period of 12 weeks may have missed the detection of long-term deep vein thrombosis (DVT) cases, potentially underestimating the true incidence and impact of DVT in the studied population.	Increase sample size to improve statistical power and generalizability. Conduct multicenter studies to minimize biases and enhance population diversity. Perform comparative studies with other joint arthroplasties (hip, knee) to assess DVT prevalence. Design prospective controlled trials to evaluate preventive interventions or treatment strategies. Extend follow-up period to capture long-term DVT cases and assess outcomes. Conduct comprehensive risk factor analysis for DVT in shoulder arthroplasty patients. Evaluate effectiveness of preventive measures (pharmacological, mechanical) for reducing DVT incidence. Include patient-reported outcomes and functional assessments in evaluations.	Willis [7]

## Data Availability

The data presented in this study are available upon reasonable request from the corresponding author. The data are not publicly available due to privacy or ethical restrictions.

## Supplementary Materials

The following supporting information can be downloaded at: https://www.mdpi.com/article/10.3390/clinpract15120221/s1: PRISMA 2020 checklist.
